# Mortality in patients with alpha-mannosidosis: a review of patients’ data and the literature

**DOI:** 10.1186/s13023-022-02422-6

**Published:** 2022-07-23

**Authors:** Julia B. Hennermann, Eva M. Raebel, Francesca Donà, Marie-Line Jacquemont, Graziella Cefalo, Andrea Ballabeni, Dag Malm

**Affiliations:** 1 Villa Metabolica, University Medical Center Mainz, Mainz, Germany; 2 Rare Disease Research Partners, Amersham, UK; 3 Chiesi Farmaceutici S.p.A., Parma, Italy; 4 Génétique Médicale, CHU La Réunion Site GHSR, Saint Pierre, France; 5 San Paolo Hospital, University of Milan, Milan, Italy; 6 Tromsø Centre of Internal Medicine, Tromsø, Norway

**Keywords:** *MAN2B1*, Alpha-mannosidosis, Mortality, Cause of death, Natural history

## Abstract

**Background:**

Alpha-mannosidosis is a rare autosomal recessive lysosomal storage disorder (LSD) caused by reduced activity of alpha-mannosidase. Clinical manifestations include skeletal dysmorphism, mental impairment, hearing loss and recurrent infections. The severe type of the disease leads to early childhood death, while patients with milder forms can live into adulthood. There are no mortality studies to date. This study aimed to investigate the age at death and the causes of death of patients with alpha-mannosidosis who had not received disease-modifying treatment.

**Methods:**

Clinicians and LSD patient organisations (POs) from 33 countries were invited to complete a questionnaire between April–May 2021. Cause of death and age at death was available for 15 patients. A literature review identified seven deceased patients that met the inclusion criteria.

**Results:**

Median age at death for patients reported by clinicians/POs was 45 years (mean 40.3 ± 13.2, range 18–56, n = 15); 53% were female. One death occurred during the patient’s second decade of life, and 14 out of 15 deaths (93.3%) during or after the patients’ third decade, including four (26.7%) during their sixth decade. Median age at death for patients identified from the literature was 4.3 years (mean 15.7 ± 17.0, range 2.2–41, n = 7); two were female. Four of the seven patients (57.1%) died within the first decade of life.

Seven of 15 deaths (46.7%) reported by clinicians/POs were recorded as pneumonia and three (20.0%) as cancer. Other causes of death included acute renal failure due to sepsis after intestinal perforation, decrease of red blood cells of unknown origin, kidney failure with systemic lupus erythematosus, aortic valve insufficiency leading to heart failure, and dehydration due to catatonia. Three out of seven causes of death (42.9%) reported in the literature were associated with septicaemia, two (28.6%) with respiratory failure and one to pneumonia following aspiration.

**Conclusions:**

This study suggests that pneumonia has been the primary cause of death during recent decades in untreated patients with alpha-mannosidosis, followed by cancer. Determining the causes of mortality and life expectancy in these patients is crucial to further improve our understanding of the natural history of alpha-mannosidosis.

## Introduction

Alpha-mannosidosis (OMIM 248500) is a rare autosomal recessive lysosomal storage disorder (LSD) caused by the deficient activity of the enzyme alpha-mannosidase owing to mutations in the *MAN2B1* gene (609458) located on chromosome 19 (19p13.13) [1]. This deficiency affects intra-lysosomal degradation pathways and leads to the progressive accumulation of undigested mannose-oligosaccharides and subsequently, impaired cell function [2, 3]. Alpha-mannosidosis is present worldwide and although its exact prevalence is not known, it is estimated at 1:500 000 live births [4]. Currently, 162 mutations of the *MAN2B1* gene are reported in The Human Gene Mutation Database [5].

Children with alpha-mannosidosis appear normal at birth, with manifestations developing at an early age and the condition progressively worsening [6]. The disorder encompasses a broad spectrum of clinical manifestations including mental and cognitive impairment, sensorineural hearing loss, facial dysmorphism, skeletal abnormalities, ataxia, motor function disturbances, immunodeficiency, recurrent infections and in older patients, behavioural problems and psychotic episodes [6–9]. Clinical phenotypes are not clearly distinguishable due to the broad heterogeneity of the disease and patients present with a continuum of clinical manifestations of varying severity which makes disease progression difficult to predict [6, 10]. Although there is no obvious correlation between genotype and clinical phenotypes [6], recent studies have indicated a relationship between *MAN2B1* genotypes and cognitive, pulmonary and motor function [11]. Even for patients with mild and moderate forms, prognosis is poor, with disease progression exerting a high impact not only on patients, but also on their families and caregivers, sometimes for several decades [12].

Alpha-mannosidosis is diagnosed by the detection of low levels of acidic alpha-mannosidase enzyme activity in peripheral blood leukocytes or cultured skin fibroblasts, with affected individuals displaying 5–15% of normal leukocyte activity [6]. To confirm the diagnosis, a molecular genetic test of pathogenic variants in *MAN2B1* should be performed [6]. However, due to the rarity and varying severity of the disease, early diagnosis can be a challenge [13] with a diagnostic delay of over a decade reported in some patients [12]. An early diagnosis is crucial to achieve best outcomes with treatment options that go beyond symptom management and supportive care [6, 10, 14]: allogeneic haematopoietic stem cell transplantation (HSCT), although there are associated risks of complications and mortality [10, 13], and enzyme replacement therapy (ERT) with velmanase alfa, which was approved in Europe in 2018 for the treatment of non-neurological manifestations in patients of mild to moderate severity [15].

Early knowledge of the natural course of the disease had been primarily based on case studies of a small number of patients. Overall clinical manifestations have been extensively described in various studies [1, 6, 9] with a limited number focusing on the characterisation of specific manifestations (e.g., cognitive function and CNS pathology [8, 16]). Some studies have reported quantitative clinical assessments (e.g., lung and cardiac function, physical endurance) and biomarkers (e.g., oligosaccharide levels) to identify clinical endpoints for study trials [2] or have predicted survival based on the modelling of clinical natural history endpoints [17].

Life expectancy for untreated patients is variable depending on disease severity and the onset of clinical manifestations. Despite a shortened life expectancy, there have been no mortality studies to date in the alpha-mannosidosis population, with causes of death and life-expectancy only mentioned as part of individual case studies. Determining the causes of death in these patients is important to improve our understanding of the clinical course of the disease, to evaluate the effectiveness of current treatment therapies, to optimise their clinical management and, ultimately, to better understand the natural history of the disease and thus improve the quality of life of patients and their families. Although an international registry collecting prospective data in treated (with ERT) and untreated patients with alpha-mannosidosis is currently ongoing [18, 19], large datasets on these patients are scarce. The aim of this study is to review the age at death and the causes of death of patients with alpha-mannosidosis as reported by clinicians and patient organisations (POs), and to review causes of death reported in published studies which include deceased patients with this disorder.

## Materials and methods

### Recruitment of clinicians and patient organisations

Seventy-eight clinicians from 70 specialised centres in 21 countries were approached, between August 2020 and May 2021, to ascertain if they held records of deceased patients with alpha-mannosidosis. Relevant clinicians were determined through the UK MPS Society, The International Advocates for Glycoprotein Storage Diseases (ISMRD), the study sponsor’s contacts, Orphanet, authors of major published studies, genetic centres, internet searches and clinicians’ referrals. In addition, 34 Rare Disease/LSD/MPS POs from 31 countries were likewise invited to participate in the study between August and November 2020. Relevant POs were identified through the UK MPS Society and ISMRD.

### Mortality in alpha-mannosidosis patients reported by clinicians and patient organisations

Data from POs and clinicians were obtained by means of a specifically designed questionnaire which was emailed to participants and completed between April and May 2021. Variables collected included: country, gender, year of birth, year of death, age at death, cause of death and *MAN2B1* mutation. The questionnaire also asked whether deceased patients had received alpha-mannosidosis therapy (e.g., HSCT, ERT) and these patients were excluded from the analysis. Other collected variables are not presented in this study. To avoid duplications while maintaining patient anonymity, patients’ initials were requested where available. The questionnaire to collect data from the UK MPS Society asked for the patient’s full name, date of birth and date of death to be able to obtain a death certificate. In the UK, a death certificate includes an exact copy of the cause of death given by a medic on the Medical Certificate of Cause of Death (MCCD) [20]. Under UK legislation, death certificates are considered public records and duplicates can be requested by anyone (Births and Deaths Registration Act 1953 [21]). Individuals were only included in the study if treatment status and cause of death were available. This research was conducted in accordance with the British Healthcare Business Intelligence Association's Legal & Ethical Guidelines for Market Research [22]. The survey study was non-interventional, and all participating POs and clinicians signed a consent form.

### Mortality in alpha-mannosidosis patients reported in the literature

A literature search was conducted in PubMed to ascertain published studies reporting on deceased patients with alpha-mannosidosis. The pre‐determined search terms ‘alpha-mannosidosis’, ‘α-mannosidosis’, ‘alpha mannosidase deficiency’ and ‘mannosidosis’ were combined using the Boolean ‘OR’ operator. Studies were retrieved if the title/ abstract/ keyword contained at least one of the terms. The Boolean term ‘NOT’ was used to exclude animal studies. Reference lists from natural history studies and reviews were hand searched for potential sources of data and subjected to the above process. Searches were completed on 21 April 2021. The Rayyan Web app [23] was used to review titles/ abstracts by one researcher to identify potential alpha-mannosidosis studies for inclusion (e.g. excluded biochemical, in vitro, molecular genetic analysis studies and those not reporting on patients). Further animal and plant studies were excluded. Full texts of relevant papers, including all case studies, were subjected to further scrutiny to include case descriptions or quantitative data of deceased patients with a genetically or laboratory confirmed diagnosis of alpha-mannosidosis (i.e. deceased patients for whom alpha-mannosidosis was only suspected were excluded, e.g. [24]) with final papers being selected based on the inclusion criteria in Table 1. Studies were examined for repeat reporting of individual patients.

**Table 1:** Inclusion and exclusion criteria of studies

Inclusion criteria	Exclusion criteria
Patients with a laboratory or genetic diagnosis of alpha-mannosidosis AND reported as deceased in the study	Full-text paper not available Not reporting the cause of death No information on the treatment status Not reporting on at least one of the following variables: year of birth, year of death or age at death

Data extraction included year of death, age at death, sex, residing country at death and cause of death where available. Individuals were only included in the study if treatment status and cause of death were available. Patients who had received HSCT or ERT were excluded from the analysis to avoid interference with the cause of death.

## Results

### Deceased patients from clinicians and patient organisations’ records

#### Responses

**Fig. 1:**
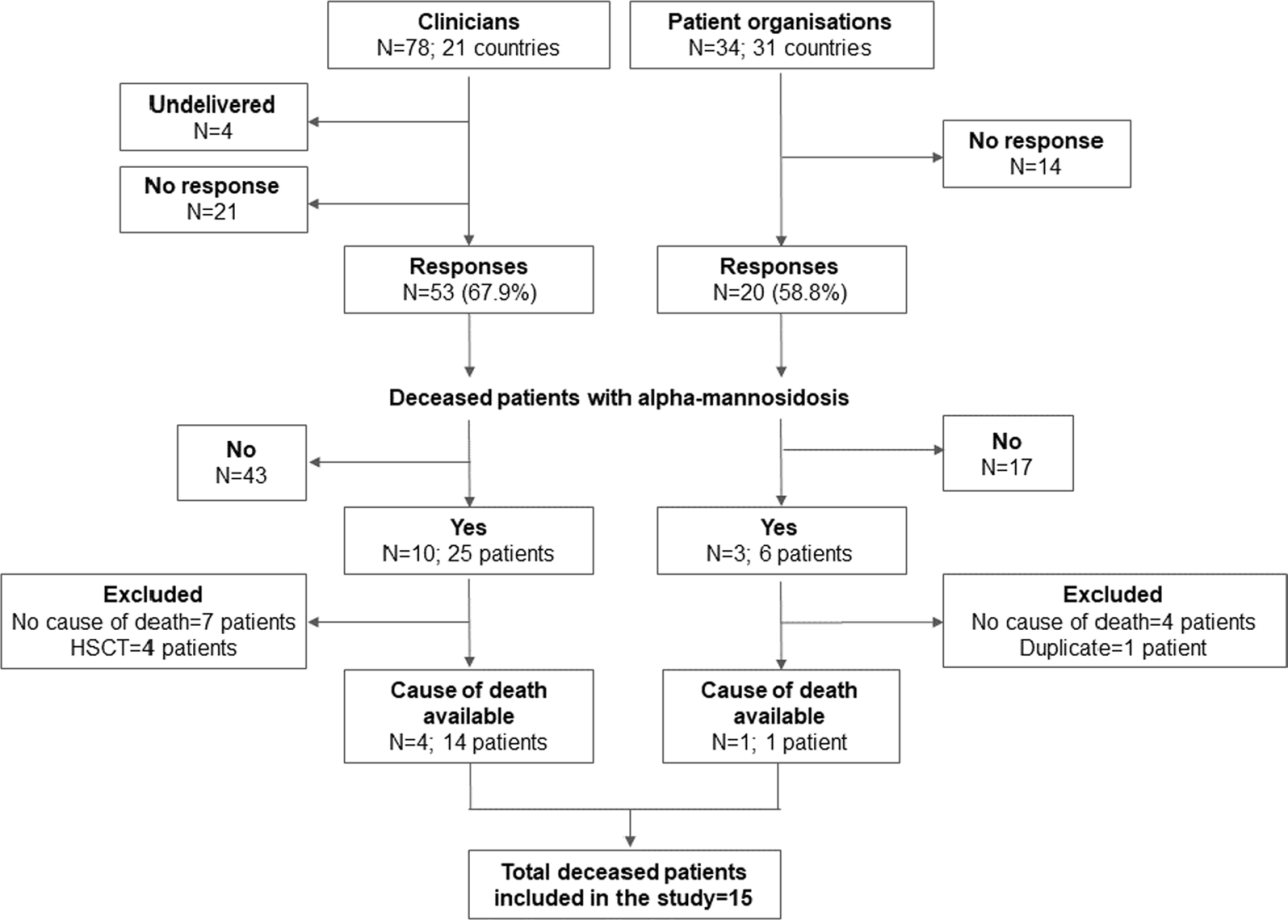
Flow diagram of responses from clinicians and patient organisations. N refers to the number of clinicians or patient organisations

A total of 15 deceased patients with a confirmed diagnosis of alpha-mannosidosis from clinicians and POs were included in the study (Fig. 1). Patient data were obtained by inviting 78 clinicians and 34 POs to participate, with responses received from 53 clinicians (response rate: 67.9%) and 20 POs (response rate: 58.8%). Ten clinicians reported 25 deceased patients with alpha-mannosidosis in their clinic or through historic records. Eleven of these patients were excluded from the analysis: seven had been lost to follow-up and the cause of death was not available and four patients died due to complications of HSCT. Fourteen deceased patients provided by four clinicians were included in the analysis (Fig. 1). Three POs had records of deceased patients on their database or knew of members that had died, with a total of six patients. Five of these patients were excluded from the study: a duplicate with a clinician’s record and four patients for whom the cause of death could not be acquired. The death certificate for the patient provided by the UK MPS Society was obtained and the cause of death was recorded (hereafter referred to as a PO record). Age at death was not available for any of the excluded patients.

#### Demographics of deceased patients from clinicians/PO’s records

Patients originated from eight different countries: France (Reunion Island) (n = 1), Germany (n = 6), Italy (n = 1), Netherlands (n = 1), New Zealand (n = 1), Norway (n = 2), UK (n = 1) and the USA (n = 2). Eight patients (53.3%) were female (Table 2). Year of birth for the 15 patients ranged from 1957 to 1992, with a median year of birth of 1970.

**Table 2:** Characteristics of deceased individuals with alpha-mannosidosis reported by clinicians/PO and the literature, for which cause of death was available

Deceased individuals reported by								
	Clinicians/PO				Literature			
	Male	Female	Unknown	Total	Male	Female	Unknown	Total
n (%)	6 (40.0)	8 (53.3)	1 (6.7)	15	4 (57.1)	2 (28.6)	1 (14.3)	7
Age at death (years)								
Median	47.0	43.5	18	45.0	28.0	3.25	2.2	4.3
Mean ± SD	44.8 ± 6.6	39.6 ± 15.1	18	40.3 ± 13.2	25.3 ± 17	3.25 ± 0.4	2.2	15.7 ± 17.0
Range	35 − 52	20 − 56	–	18 − 56	4.3 − 41	3 − 3.5	–	2.2 − 41
Age at death, n (%)								
0–9	–	–	–	–	1 (25)	2 (100.0)	1 (100)	4 (57.1)
10–19	–	–	1 (100)	1 (6.7)	1 (25)	–	–	1 (14.3)
20–29	–	3 (37.5)	–	3 (20.0)	–	–	–	–
30–39	2 (33.3)	1 (12.5)	–	3 (20.0)	1 (25)	–	–	1 (14.3)
40–49	2 (33.3)	1 (12.5)	–	4 (26.7)	1 (25)	–	–	1 (14.3)
≥ 50	1 (16.7)	3 (37.5)	–	4 (26.7)	–	–	–	–

#### Mortality in patients from clinicians/PO records

**Fig. 2:**
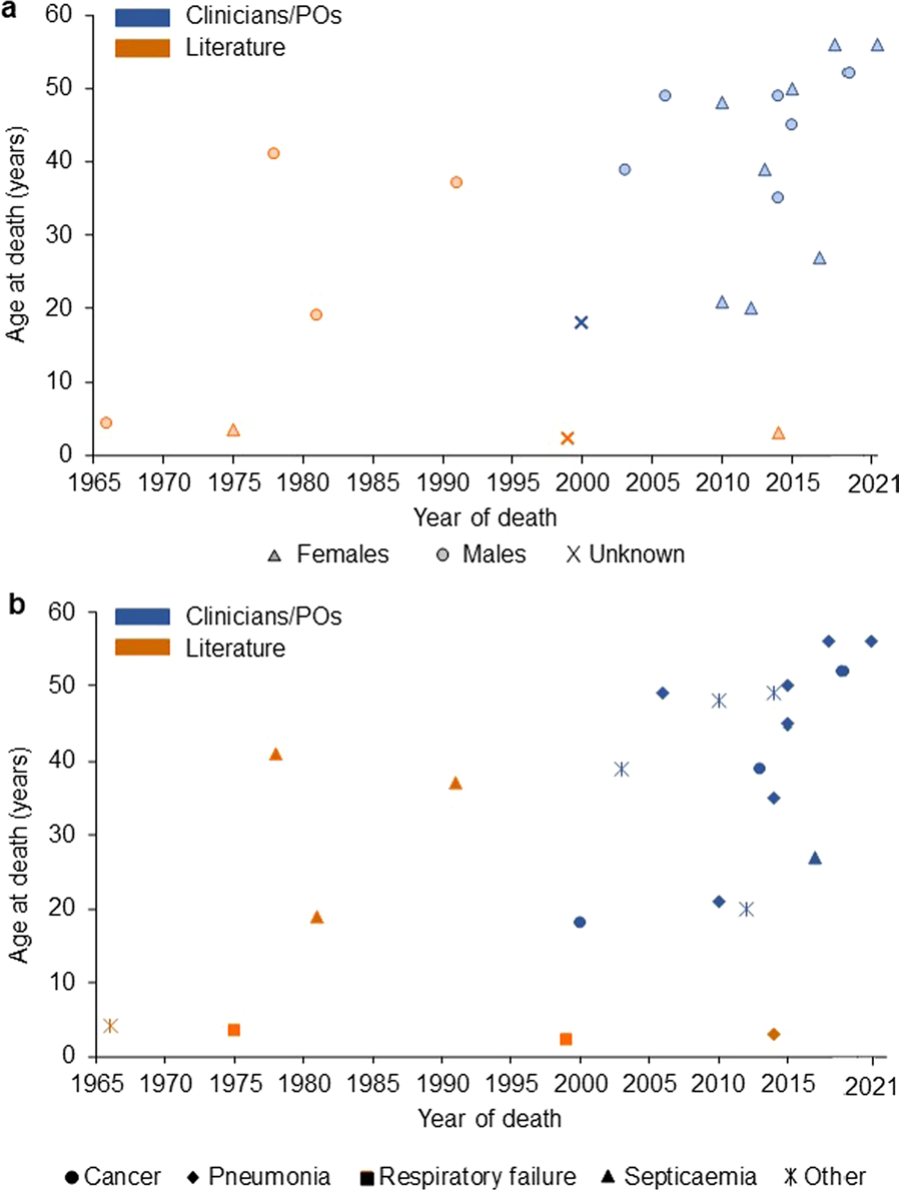
Year of death and age at death (years) of patients from clinicians/POs (blue) and the literature (orange) by (**a**) data source and sex and (**b**) data source and cause of death

Median age at death of the 15 patients was 45 years (mean 40.3 ± 13.2, range 18–56), with a median age at death of 47 years for males and 43.5 years for females (Table 2). Death records ranged between the years 2000 and 2021. The majority of patients (80.0%, n = 12) died between the years 2010–2021 (Fig. 2a), including all females (Fig. 2a). One death was recorded during the patient’s second decade of life (i.e., 10–19 years of age) but 14 out of 15 deaths (93.3%) occurred during or after the patients’ third decade (i.e., ≥ 20 years of age), including four deaths (26.7%) recorded during the patients’ fifth decade (40–49 years of age) and four (26.7%) during the sixth decade (≥ 50 years of age) (Table 2; Fig. 2a). Two female patients reached the maximum age of 56 years (Fig. 2a).

Out of the 15 patients, seven deaths (46.7%) were associated with pneumonia, including one death due to aspiration pneumonia and one death due to pneumonia reported together with cachexia (Fig. 2b; Table 3). Median age of patients who died from pneumonia was 49 years (mean 44.6 ± 12.6, range 21–56) and four were female. Three deaths (20%) were associated with cancer, i.e., colon carcinoma, breast cancer and leukaemia (Fig. 2b; Table 3). Median age at death of patients who died from cancer was 39 years (mean 36.3 ± 17.2; range 18–52). One female patient died from septicaemia after intestinal perforation, leading to acute renal failure at the age of 27 years. Other causes of death are shown in Table 3. Genetic mutations confirming a diagnosis of alpha-mannosidosis were available for twelve patients and are additionally included in Table 3.

**Table 3:** Conditions reported by clinicians/PO as a cause of death in fifteen patients with alpha-mannosidosis

Patient Cause of death		n (%)	Sex	Year of death	Age at death (years)	Mutations identified in *MAN2B1*; cDNA p.(protein)
	**Pneumonia**	7 (46.7)				
1	Pneumonia		M	2006	49	n/a
2	Pneumonia		F	2010	21	n/a
3	Pneumonia		M	2014	35	c.2402delG p.(G2801AfsX4); c.2665-42_2665-77del36; c.2665-5_2665-39del35; c.2665-5_2665-39del35
4	Pneumonia, aspiration		M	2015	45	c.1831-2A > G p.(His611Glyfs*3); c.1831-2A > G p.(His611Glyfs*3)
5	Pneumonia, with pleura empyema and pneumothorax		F	2015	50	c.2175_2177delins11 p.(Trp725CysfsX4); c.2175_2177delins11 p.(Trp725CysfsX4)
6	Cachexia, pneumonia		F	2018	56	c.1816delA p.(Thr606ProfsX18); c.1830 + 1G > C(IVS14 + 1G > C)
7	Pneumonia		F	2021	56	c.685C > T p.(Arg229Trp); c.1830 + 1G > C p.(Val549_Glu610del)
	**Septicaemia**	1 (6.7)				
8	Acute renal failure due to sepsis, after intestinal perforation due to inflammatory bowel disease		F	2017	27	c.788C > T p.(Pro263Leu); c.2355G > A p.(Arg757Metfs*6)
	**Cancer**	3 (20.0)				
9	Leukaemia		n/a	2000	18	n/a
10	Breast cancer		F	2013	39	c.1830 + 1G > C (IVS14 + 1G > C); c.1830 + 1G > C(IVS14 + 1G > C)
11	Colon carcinoma		M	2019	52	n/a p.(Tyr99ValfsX61); n/a p.(Tyr99ValfsX61)
	**Other**	4 (26.7)				
12	Dehydration due to catatonia during a psychotic episode		M	2003	39	c.1026 + 2 T > G p.(Gln339_Val342del4); c.1026 + 2 T > G p.(Gln339_Val342del4)
13	Kidney failure, systemic lupus erythematosus		F	2010	48	c.1831-2A > G p.(His611Glyfs*3); c.1831-2A > G p.(His611Glyfs*3)
14	Aortic valve insufficiency, heart failure		F	2012	20	c.1109G > A p.(Trp370*); c.1109G > A p.(Trp370*)
15	Decrease of red blood cells (deglobulisation) of unknown origin		M	2014	49	c.2165 + 1G > A (IVS17 + 1G > A); c.2165 + 1G > A (IVS17 + 1G > A)

F = female, M = male, n/a = not available

### Deceased patients with alpha-mannosidosis reported in the literature

A total of 392 studies were identified in the PubMed search and were subjected to abstract review; of these, 316 were excluded. Full-text review was performed on 76 papers, and 64 were further excluded according to the pre-determined inclusion/exclusion criteria (Table 1). Twelve studies reported information on ten deceased patients, including causes of death: one study had two deceased patients [10], one patient was described within two studies [25, 26] and another patient was described within three studies [27–29]. Details for these patients had been described as individual case reports, case series or aggregated data. Three patients were excluded from the analysis as their deaths had occurred post-HSCT transplant [10, 30]. The authors of Zielonka et al. [17] included aggregated causes of death in alpha-mannosidosis patients in their supplementary information and were contacted to compare findings. All of the reports used in their study had been found by our literature search but only those for which a cause of death had been explicitly recorded within the publication were included in our study. A total of seven deceased patients from the literature, with a confirmed diagnosis of alpha-mannosidosis, were included in our study (Table 4).

**Table 4:** Conditions reported in the literature as a cause of death in seven patients with alpha-mannosidosis

Patient	Cause of death	n (%)	Sex	Age at death (years)	Study
	**Pneumonia**	1 (14.3)			
1	Bacterial pneumonia following aspiration		F	3.0	[31]
	**Septicaemia**	3 (42.9)			
2	*Klebsiella pneumoniae* septicaemia		M	19.0	[32]
3	*Beta-haemolytic streptococci* septicaemia and renal failure		M	41.0	[33]
4	Septicaemia after numerous pulmonary and cutaneous infections		M	37.0	[34]
	**Respiratory failure**	2 (28.6)			
5	Respiratory failure		n/a	2.2	[35]
6	Respiratory failure and disseminated intravascular coagulation		F	3.5	[25, 26]
	**Other**	1 (14.3)			
7	Ketoacidosis and dehydration, suspected intracranial pressure		M	4.3	[27–29]

F = female, M = male, n/a = not available

Studies were published in six countries: France (Reunion Island) (n = 1), Italy (n = 1), South Africa (n = 1), Sweden (n = 1), UK (n = 1) and the USA (n = 2). Two (28.6%) of the seven patients were female (Table 2; Fig. 2b). Median age at death of the seven patients was 4.3 years (mean 15.7 ± 17.0, range 2.2–41). The median year of death was 1981, with records of death ranging between the years 1966 to 2014. Four out of the seven patients (57.1%) died within the first decade of life (i.e., 0–9 years) and all patients but one (n = 6) were deceased before 1999 (Fig. 2a).

Three causes of death (42.9%) were associated with septicaemia: *Klebsiella pneumoniae* septicaemia, *Beta-haemolytic streptococci* septicaemia leading to renal failure, and septicaemia after numerous pulmonary and cutaneous infections (Table 4; Fig. 2c). Studies reported two patients with respiratory failure as a cause of death, with one patient described as deceased due to respiratory failure and disseminated intravascular coagulation. One study recorded pneumonia following aspiration as the cause of death. Causes of death reported in the literature are shown in Table 4.

## Discussion

This is the first retrospective study of individuals with alpha-mannosidosis reporting on the causes of death and the age at death in these patients. Death records from clinicians/PO suggested that pneumonia was the most prevalent (46.7%) cause of death in our population over the past fifteen years. Respiratory, gastrointestinal and ear infections are a recurrent feature during the first decade of life in patients with alpha-mannosidosis, with frequency of infections, including pneumonia, decreasing after the second decade [1, 2, 9], however, the mean age of patients dying from pneumonia in our study was 49 years (died in the third-fourth decades = 2; fifth-sixth decades = 5), suggesting patients remain susceptible to infections throughout their lives.

A compromised immune response to infections may also explain the high incidence of bacterial infections and sepsis in this population and in the literature. There is evidence for immunodeficiency in alpha-mannosidosis patients, with children with alpha-mannosidosis who had been vaccinated against tetanus, diphtheria and poliovirus showing significantly lower levels of serum antibodies than the normal population [7], suggesting antibody levels in these patients may also be deficient against other infections. Malm et al. [7] found that, in alpha-mannosidosis patients, the density of neutrophil receptors involved in phagocytosis did not increase in the presence of autologous serum. This may indicate an altered immunological response with a phagocytic deficiency and a compromised intracellular killing capacity in the presence of bacterial infections. Indeed, besides pneumonia, our study reported a patient who died from sepsis after intestinal perforation, and the literature described three individuals who died from sepsis and one from bacterial pneumonia. Nevertheless, it is not known from our data if most records of pneumonia were a consequence of bacterial infections or of swallowing problems associated with functional decline as patients get older, which may result in aspiration pneumonia. Pneumonia and respiratory complications have been found to be a leading cause of mortality in other LSDs also involving progressive neurological impairment [36–39].

A descriptive study on the natural disease course of patients with alpha mannosidosis reported patients presenting with autoimmune conditions such as pancytopenia, systemic lupus erythematosus (SLE), or primary biliary cirrhosis [9]. In our study, a clinician reported SLE and kidney failure as the causes of death of a 48 year of female, however the clinical pathway leading to kidney failure is unknown from the available records.

Three of the fifteen individuals in our study population died from cancer: breast cancer (39 years old), colon carcinoma (52 years old) and leukaemia (18 years old). The risk of breast and colorectal cancer increases with age [40, 41] but our small sample size and the lack of information about additional risk factors hinders a direct comparison with non-alpha-mannosidosis populations. However, an increased risk of cancer has been found for other LSDs suggesting that impaired lysosomal activity in alpha-mannosidosis may also contribute to carcinogenesis. Gaucher disease has been associated with an increased incidence of multiple myeloma, hepatocellular carcinoma and lymphocytic leukaemia [42–44], a raised prevalence of cancer has been described among patients with Niemann-Pick disease type A and B [45] and Niemann-Pick disease type C1 has been associated with an increased risk of hepatocellular carcinoma [46]. A review of cancer cases in Fabry disease patients showed an increased incidence for specific cancers, such as melanoma, urological malignancies and meningiomas [47]. Analysis of data from international sequencing projects to elucidate the association between cancer and germline variants in causal genes of LSDs found that carriers of potentially pathogenic variants in LSD genes were at an increased risk of cancer [48]. Currently, the association between cancer and alpha-mannosidosis is not understood and warrants further study to elucidate the mechanisms leading to cancer in these patients.

Behavioural problems and psychosis have been described as increasing with age in alpha-mannosidosis patients and they have been recorded in 70% of patients over the age of 30 [9]. In our study, a 39 year old patient died from dehydration caused by catatonia during a psychotic episode. The literature also mentioned a 4 year old child who died from ketoacidosis and dehydration but it is not known if this was caused by intracranial pressure or by violent restlessness due to psychiatric disease.

A quantitative analysis of 111 published cases revealed 72% of patients with alpha-mannosidosis were still alive after the age of 41 [17], a similar pattern to our clinicians/PO population where 53% of individuals were still alive after the age of 40. The only three children reported by clinicians had died by the time they were seven years old but, together with a paediatric patient described in the literature, were excluded from our study because they had undergone HSCT. In all cases, death was the result of complications post-HSCT transplant. Although long-term experience with HSCT in alpha-mannosidosis has not yet been determined, a follow-up study of 17 patients treated with HSCT at a median age of 3.6 years found 15 were still alive after 5.5 years [10].

It was difficult to compare differences in the causes of death and the age at death reported by the clinicians/PO and the literature due to an increased awareness and understanding of the disease and the recent availability of diagnostic tools. There were considerable differences in the year of death and age at death between the groups with the age of the deceased population provided by clinicians/PO averaging 40 years at the time of death versus an average of 16 years in the literature. This age difference may be due to the limited knowledge of the disease that was available at the time these early studies were published, implying that early descriptions may only include the most severe and obvious forms of the disease, while older and milder patients may have been misdiagnosed or not diagnosed at all as no genetic testing was available at the time. It is therefore impossible from our study to determine whether earlier diagnosis and improved healthcare have had an influence on patients living longer. In addition, the patient population provided by clinicians/POs did not include the more severely affected patients with early disease onset, which would have caused an early childhood death. Therefore, we are not able to determine if the causes of death found in our population would also be prevalent within the more severely affected patients.

A limitation of this study was the small sample size and highlights the difficulty of retrieving records of deceased patients with a rare disease. Some deceased patients had been lost to follow up (e.g., death had occurred in another hospital). Two POs informed us that they were obliged to delete details of deceased members from their database due to information privacy policies. In other cases, POs knew about deceased members but had not recorded any further information on their database.

## Conclusion

Our study suggests that pneumonia, whether caused by a compromised immunity or due to other causes such as functional deterioration, has been the primary cause of death over the recent decades in these patients, followed by cancer. Determining the causes of mortality and life expectancy in patients with alpha-mannosidosis is paramount to further improve our understanding of the natural history of the disease. Moreover, alpha-mannosidosis is still underdiagnosed despite current improvements in genetic testing [49]. A better understanding of the natural history of alpha-mannosidosis and its associated comorbidities may support an earlier diagnosis and, consequently, allow for timely interventions to manage these patients.

## Data Availability

The datasets generated and/or analysed during the current study are not publicly available due to patient confidentiality.
